# Stents migration into right atrium from severely calcified superior vena cava in a hemodialysis patient

**DOI:** 10.1016/j.heliyon.2023.e23621

**Published:** 2023-12-13

**Authors:** Wei Wei, Qiuyan Zhao, Caihong Liu, Letian Yang, Jian Li, Ping Fu, Yuliang Zhao, Tianlei Cui

**Affiliations:** a Division of Nephrology and Kidney Research Institute, West China Hospital, Sichuan University, Chengdu, China; b Outpatient Department, West China Hospital of Sichuan University/West China School of Nursing, Sichuan University, Chengdu, China

**Keywords:** Case report, SVC, Stent, Migration, Hemodialysis

## Abstract

Vascular calcification is common among hemodialysis patients. In this report, we presented a case of superior vena cava (SVC) stent migration during endovascular angioplasty in a 50-year-old female hemodialysis patient with severe SVC calcification. The stent migration was refractory to the deployment of a second anchor stent, which shortly resulted in pericardium tamponade and was successfully rescued by emergent thoracotomy. The potential role of vascular calcification as a risk factor to stent migration was discussed. Patients with severe vascular calcification receiving endovascular angioplasty might need a careful risk screening for stent migration.

## 1 Introduction

Endovascular angioplasty is widely applied in hemodialysis patients complicated with central venous stenosis (CVS). Stent migration is an uncommon but potentially fatal complication of central vein angioplasty, with a reported incidence of 2–3% [[1], [2], [3], [4]]. Calcified vessel recoils poorly, providing less elasticity against the stents, and preventing stent incorporation into the vessel wall [5]. Hemodialysis patients have a high prevalence of vascular calcification [6], especially in those with poorly controlled mineral and bone disorder, who might need a different strategy in the prevention and management of stent migration.

## 2 Case presentation

**Fig. 1:**
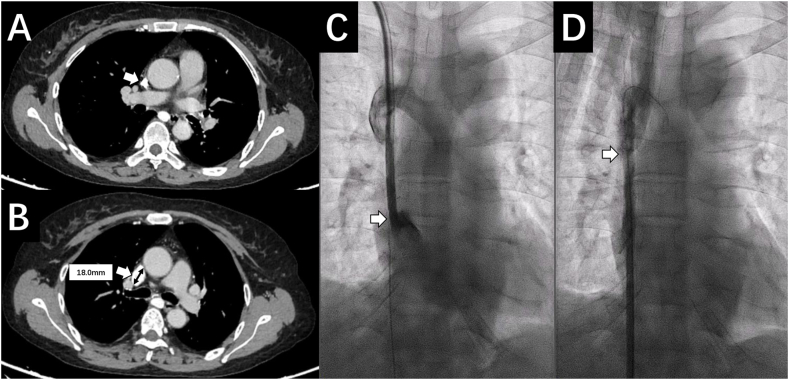
A: Superior vena cava calcification and stenosis by CT venography, white arrow: superior vena cava calcification and stenosis. B: Measurement of superior vena cava diameter, white arrow: superior vena cava diameter was estimated to be about 18.0 mm. C: Superior vena cava stenosis by DSA, white arrow: proximal remnant of superior vena cava. D: Superior vena cava stenosis by DSA, white arrow: distal remnant of superior vena cava.

A 50-year-old female patient who had been on maintenance hemodialysis for 10 years was admitted for tunneled cuffed central venous catheter (tcCVC) dysfunction for 2 months. 10 years ago, the patient started hemodialysis with a left forearm arteriovenous fistula (AVF) which lasted for 5 years, followed by 2 unsuccessful attempts of AVF creation on the left and right forearm respectively. The patient had since been using a right neck tcCVC. Upon admission, the patient was temporarily dialyzed through a femoral catheter. Her vital signs were unremarkable. Physical examination revealed facial edema, upper extremities swelling and extensive varicose on thoracic wall. Laboratory tests were remarkable for hemoglobin 110g/L, serum creatinine 485μmol/L, C-reactive protein 13mg/L, parathyroid hormone 47pmol/L (448pg/mL), Phosphate 2.47mmol/L, and Calcium 2.52mmol/L. CTvenography indicated stenosis and calcification of superior vena cava (SVC) (Fig. 1 A). To relieve her symptoms of SVC syndrome and make possible for AVF or arteriovenous graft (AVG) creation as per KDOQI guideline [7], we decided to dilate/stent her SVC and restore its venous flow. The stent size was decided with reference to the adjacent normal SVC basing on preprocedural CT, showing the diameter of SVC to be 18.0 mm (Fig. 1 B). The stent diameter is supposed to be 10 %–20 % or 1–2 mm larger than the venous diameter to prevent stent migration, so we chose the 20 mm diameter stent.

**Fig. 2:**
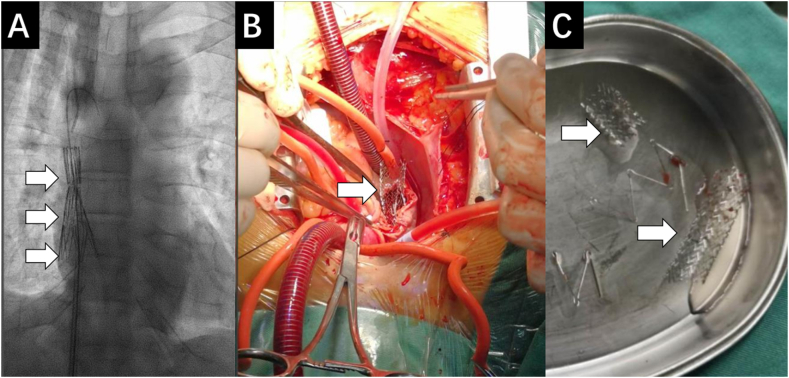
A: Stents migrated into right atrium by DSA. B: Thoracotomy and removal of stents from right atrium. C: Stents were successfully removed. White arrow: stents.

SVC stenosis was confirmed by Digital Subtraction Angiography (DSA) (Fig. 1C and D). With patient's inform consent, the dysfunctional tcCVC was firstly removed. After percutaneous graded balloon dilation (Synergy balloon catheter, Boston Scientific, Natick, MA, USA; 6 mm*4.0 cm, 10mm*4.0 cm), a self-expanding Z-stent (Boston Scientific, Natick, MA, USA; 20 mm*6.0 cm) was implanted. The position of the stent was confirmed immediately after deployment under DSA, which was however found to be migrating slowly towards the heart. Another bare stent (12 mm*6.0 cm) was released trying to anchor the first stent. Unfortunately, both stents kept migrating until they eventually reached the right atrium (Fig. 2 A). The patient presented with tachycardia (135/min), cardiac shock (BP 54/32 mmHg) and unconsciousness shortly and was intubated. Transthoracic echocardiogram showed pericardium tamponade, while transesophageal echocardiogram further confirmed stents displacement into the right atrium, with the proximal end reaching tricuspid orifice and atrial septum. Left to right shunt could be detected at the atrial septum level, as well as thrombus formation in the right atrium.

Emergency pericardium puncture and thoracotomy were performed. Stents could be palpated at SVC and right atrium, with the proximal edge trapped into the opening of inferior vena cava, tricuspid valve orifice and atrial septum. Extensive vascular calcification was found in SVC and right brachiocephalic vein. On cardiopulmonary bypass, the surgeons successfully removed the stents and thrombus in the right atrium as well as the pericardium hematoma (Fig. 2B and C). Right atrial wall and atrial septum perforations were also repaired. After the operation, the patient stabilized and BP increased to 95/65 mmHg. Afterwards, the patient was implanted with a new tcCVC under DSA guidance and successfully discharged 16 days later. During follow-up, the patient was referred to vascular surgeons for the evaluation of AVF establishment. Informed consent to report the case was obtained from the patient.

## 3 Discussion

Stent migration is a rare but potentially life-threatening complication in patients who received interventional angioplasty for CVS. The Society of Vascular Surgery defines stent migration as a movement of ≥10 mm relative to anatomical landmarks or any migration leading to symptoms or requiring treatment. Stent migration has been reported occasionally in the literature and arouses the attention by health-care providers. As early as 1996, Entwisle and colleagues reported a stent migration complicating the treatment of malignant SVC stenosis [8], followed by more recent cases of stent migration into right atrium, right ventricle, and pulmonary artery [[9], [10], [11]].

Stent migration could end up with various outcomes. In some situations, displaced stents could be left in-situ with no disastrous consequences. But for most cases, depending on the location and clinical manifestation, migrated stents should be either properly fixed to another stent to avoid further dislodgement [12], or retrieved to prevent vessel trauma, valves/papillary muscle injury, and rarely, myocardial perforation [13]. Percutaneous removal of intravascular foreign bodies has been well accepted as a safe and successful management option [14]. However, when a stent is transversely stuck in the vessel lumen or heart chamber, or mechanical injury is evident, open surgery might serve as the last resort. Slonim and colleagues summarized their experience in the management of 27 misplaced or migrated endovascular stents. While most stents were successfully managed percutaneously, one stent was eventually surgically removed from the right ventricle due to tricuspid insufficiency [1]. In the present case, our first attempt to anchor the migrating stent with a second stent failed. As both stents finally dropped into the right atrium causing cardiac shock and pericardium tamponade, thoracotomy and open surgical repair were clearly indicated for the rescue. Unlike late (postoperative) migrations previously reported, immediate migratory stents, as in this case, could make the way unimpeded to the heart and lungs shortly, warranting urgent management.

The risk factors of stent migration were thoroughly summarized by Sequeira et al., including anatomy of veins, hemodynamic forces, stent properties and decision-making errors [15], however the risk factors for immediate or late stent migrations might be different, and remain to be further investigated. In hemodialysis patients, the deformity of central venous system (such as multiple stenoses) could result in a poor vein-stent apposition, thus promoting migration. The high-volume blood flow in patients with a working arteriovenous fistula also contributes as a driving force in the long term. Under-sizing of stents is the most common operator error for stent migration. Careful measurement of central vein remnant is needed for the selection of stents with suitable length and diameter. As shortening and radial recoil of stents had been reported [16], the stent diameter is supposed to be 10 %–20 % or 1–2 mm larger than the venous diameter to prevent stent migration [15,17]. In dialysis patients, the fibrotic and calcified vessel wall is especially prone to laceration during over-dilation. A compromise sometimes might need to be made between adequate stent size and risk of vessel laceration.

Vascular calcification, as a consequence of poorly controlled hyperparathyroidism and calcium-phosphate metabolic disorder in end-stage renal disease patients, involves both large and small vessels [6]. Other reported risk factors of vascular calcification include increasing age, male gender, dialysis vintage, hyperphosphatemia, a positive net calcium and phosphate balance, as well as excessive oral calcium intake [[18], [19], [20]]. Calcified vasculature recoils poorly, providing less elasticity against the stents, and preventing stent incorporation into the vessel wall [5], which might explain for the high migration tendency in the current case. Similarly, in a prospective cohort analysis in patients receiving endovascular aneurysm repair, intraoperative stent graft rotation was strongly associated with higher levels of iliac artery calcification [21]. We therefore hypothesized that in hemodialysis patients, central venous calcification might be a risk factor for stent migration. Quantitative analysis of vascular calcification may also facilitate risk stratification of stent migration during endovascular procedures. To our knowledge, there are no commercially available stents that are designed for vessels with severe calcification. Stents that generate greater friction on calcified intima might be a better choice for these patients so as to prevent stent migration.

## 4 Conclusion

We presented a hemodialysis patient with vascular calcification who encountered immediate SVC stents migration into right atrium, ending up with intraoperative pericardium tamponade and rescued by emergent thoracotomy. Calcification diminishes vessel elasticity and might be a risk factor to stent migration. More clinical studies are required to verify this hypothesis, as well as to provide insight into the development of next generation of stent suitable for patients with severe vascular calcification. This case also underlines the importance of multidisciplinary cooperation and the availability of open-heart surgery in interventional nephrology practice.

## Ethics statement

Institutional Review Board approval was not required as this was a case report.

## Informed consent statement

Informed consent to report the case was obtained from the patient.

## Additional information

No additional information is available for this paper.

## Funding statement

This study was supported by 10.13039/501100012166National Key Research and Development Program of China (2023YFC2411805), Sichuan University (2023SCUH0065) and 135 Project for Disciplines of Excellence, 10.13039/501100013365West China Hospital, Sichuan University (2020HXFH014). The funding sources had no involvement in this study.

## Data availability statement

Data will be made available on request. The research data presented in the study are included in the article. Further inquiries can be directed to the corresponding authors.

## CRediT authorship contribution statement

**Wei Wei:** Writing – review & editing, Writing – original draft, Investigation. **Qiuyan Zhao:** Writing – review & editing, Writing – original draft, Investigation. **Caihong Liu:** Writing – review & editing, Methodology, Investigation, Data curation. **Letian Yang:** Writing – review & editing, Methodology, Investigation, Data curation. **Jian Li:** Writing – review & editing, Methodology, Investigation, Data curation. **Ping Fu:** Writing – review & editing, Data curation, Conceptualization. **Yuliang Zhao:** Writing – review & editing, Methodology, Investigation, Data curation, Conceptualization. **Tianlei Cui:** Writing – review & editing, Writing – original draft, Methodology, Investigation, Data curation.

## Declaration of competing interest

None.

## References

[1] Slonim S.M., Dake M.D., Razavi M.K., Kee S.T., Samuels S.L., Rhee J.S., Semba C.P. (1999). Management of misplaced or migrated endovascular stents. J. Vasc. Intervent. Radiol. : JVIR..

[2] Gabelmann A., Kramer S.C., Tomczak R., Gorich J. (2001). Percutaneous techniques for managing maldeployed or migrated stents. J. Endovasc. Ther. : an official journal of the International Society of Endovascular Specialists.

[3] Dashkoff N., Blessios G.A., Cox M.R. (2010). Migration of covered stents from hemodialysis A-V access to the pulmonary artery: percutaneous stent retrieval and procedural trends. Cathet. Cardiovasc. Interv. : official journal of the Society for Cardiac Angiography & Interventions.

[4] Eguchi D., Honma K. (2020). Results of stenting for central venous occlusions and stenoses in the hemodialysis patients. Annals of vascular diseases.

[5] Sequeira A., Abreo K. (2014). The structure and function of endovascular stents: a primer for the interventional nephrologist. Semin. Dial..

[6] Kraus M.A., Kalra P.A., Hunter J., Menoyo J., Stankus N. (2015). The prevalence of vascular calcification in patients with end-stage renal disease on hemodialysis: a cross-sectional observational study. Therapeutic advances in chronic disease.

[7] Lok C.E., Huber T.S., Lee T., Shenoy S., Yevzlin A.S., Abreo K., Allon M., Asif A., Astor B.C., Glickman M.H., Graham J., Moist L.M., Rajan D.K., Roberts C., Vachharajani T.J., Valentini R.P. (2020). KDOQI clinical practice guideline for vascular access: 2019 update. Am. J. Kidney Dis. : the official journal of the National Kidney Foundation.

[8] Entwisle K.G., Watkinson A.F., Reidy J. (1996). Case report: migration and shortening of a self-expanding metallic stent complicating the treatment of malignant superior vena cava stenosis. Clin. Radiol..

[9] Anand G., Lewanski C.R., Cowman S.A., Jackson J.E. (2011). Superior vena cava stent migration into the pulmonary artery causing fatal pulmonary infarction. Cardiovasc. Intervent. Radiol..

[10] Khalid M.O., Moskovits N., Frankel R.A., Moskovits M., Saunders P.C., Jacobowitz I.J., Ribakove G.H., Shani J. (2020). Venous stent migrating to the right heart causing severe regurgitation. Journal of investigative medicine high impact case reports.

[11] Orellana-Barrios M., Patel N., Arvandi A., Paone R., Santana D. (2017). Venous stent migration into right ventricle. Cureus.

[12] Gabelmann A., Kramer S., Gorich J. (2001). Percutaneous retrieval of lost or misplaced intravascular objects. AJR American journal of roentgenology.

[13] Poludasu S.S., Vladutiu P., Lazar J. (2008). Migration of an endovascular stent from superior vena cava to the right ventricular outflow tract in a patient with superior vena cava syndrome. Angiology.

[14] Haddad P.G., Mohamed A., Peden E.K. (2021). Novel technique of stent retrieval after migration to the right heart. Journal of vascular surgery cases and innovative techniques.

[15] Sequeira A. (2016). Stent migration and bail-out strategies. J. Vasc. Access.

[16] Vesely T.M., Hovsepian D.M., Pilgram T.K., Coyne D.W., Shenoy S. (1997). Upper extremity central venous obstruction in hemodialysis patients: treatment with Wallstents. Radiology.

[17] Siani A., Marcucci G., Accrocca F., Antonelli R., Mounayergi F., Rosati M.S., Gabrielli R. (2012). Endovascular central venous stenosis treatment ended with superior vena cava perforation, pericardial tamponade, and exitus. Ann. Vasc. Surg..

[18] Aaltonen L., Koivuviita N., Seppänen M., Kröger H., Tong X., Löyttyniemi E., Metsärinne K. (2022). Association between bone mineral metabolism and vascular calcification in end-stage renal disease. BMC Nephrol..

[19] Cozzolino M., Ciceri P., Galassi A., Mangano M., Carugo S., Capelli I., Cianciolo G. (2019). The Key role of phosphate on vascular calcification. Toxins.

[20] Wu P.Y., Lee S.Y., Chang K.V., Chao C.T., Huang J.W. (2021).

[21] Crawford S.A., Sanford R.M., Doyle M.G., Wheatcroft M., Amon C.H., Forbes T.L. (2018). Prediction of advanced endovascular stent graft rotation and its associated morbidity and mortality. J. Vasc. Surg..

